# *SPR9* encodes a 60 S ribosomal protein that modulates panicle spreading and affects resistance to false smut in rice (*Oryza sativa*. L)

**DOI:** 10.1186/s12870-023-04172-4

**Published:** 2023-04-20

**Authors:** Niqing He, Fenghuang Huang, Libin Lu, Xun Wang, Qingshun Q. Li, Dewei Yang

**Affiliations:** 1 Rice Research Institute, Fujian Academy of Agricultural Sciences, Fujian High Quality Rice Research and Development Center, Fuzhou, 350019 Fujian China; 2 Biomedical Sciences, College of Dental Medicine, Western University of Health Sciences, Pomona, CA 91766 USA

**Keywords:** Rice (*Oryza sativa L.)*, *Spreading panicle* mutant, Gene cloning, Rice false smut (RFS)

## Abstract

**Background:**

The architecture of inflorescence in crops is a key agronomic feature determining grain yield and thus has been a major target trait of cereal domestication.

**Results:**

In this study, we show that a simple spreading panicle change in rice panicle shape, controlled by the *Spreading Panicle 9* (*SPR9)* locus, also has a significant impact on the resistance to rice false smut (RFS). Meanwhile, we mapped a novel *spr9* mutant gene between markers Indel5-18 and Indel5-22 encompassing a genomic region of 43-kb with six candidate genes. Through gene prediction and cDNA sequencing, we confirmed that *LOC_Os05g38520* is the target gene in the *spr9* mutant, which encodes 60 S ribosomal protein L36-2. Further analysis showed that the *spr9* mutant is caused by a 1 bp deletion in the first exon that resulted in premature termination. Knockout experiments showed that the *SPR9* gene is responsible for the spreading panicle phenotype of the *spr9* mutant. Interestingly, the *spr9* mutant was found to improve resistance to RFS without affecting major agronomic traits. Taken together, our results revealed that the *spr9* allele has good application prospects in rice breeding for disease resistance and panicle improvement.

**Conclusions:**

We report the map-based cloning and functional characterization of *SPR9*, which encodes a 60 S ribosomal protein that regulates spreading panicles and affects the resistance to false smut in rice.

**Supplementary Information:**

The online version contains supplementary material available at 10.1186/s12870-023-04172-4.

## Background

Plant inflorescence configuration is a key agronomic factor affecting mine grain yield and a major target for crop domestication and improvement [1]. Understanding the genetic basis of crop inflorescence structure will not only help to clarify the evolutionary mechanism of crops but also help to improve crop yield.

Rice (*Oryza sativa. L*) is one of the most important food crops in the world, and its panicle morphological development and molecular regulation mechanism have been the focus of research. In recent years, some progresses have been made in the study of panicle development of rice, a monocot model plant, but it is far less detailed than that *Arabidopsis thaliana* [2]. The panicle formation process of rice is a complex physiological and biochemical process involving axillary meristem development, inflorescence structure building and grain development. The in-depth study of panicle formation will not only help to reveal the regulatory mechanism of panicle morphogenesis but also provide theoretical guidance for the improvement of panicle type in rice [3]. Genes related to rice panicles, such as *Gn1a/OsCKX2* [4], *DEP1* [5, 6], *GNP1/GA20ox1* [7], *IPA1/WFP/OsSPL14* [8, 9], *NPT1/OsOTUB1* [10], *GS3*[11], *TGW6*[12], *GW8/OsSPL16* [13], *GW7/GL 7* [14], *GLW7/OsSPL13* [15], *GS2/GL2/OsGRF4* [16–18], *GW2* [19], and *GW5* [20], have been successfully cloned.

Transition from a spread panicle typical of ancestral wild rice (*Oryza rufipogon* Griff.) to the compact panicle of present cultivars (*Oryza sativa L*.) was a crucial event in rice domestication [21]. In recent years, a number of panicle spreading-related genes or QTLs were mapped using different genetic populations, such as *spr1* [22], *spr2* [23], *spr3* [24], *spr4* [25], *spr5* [26], *spr8* [27] and *OsLG1* [1]. However, of these localized genes or QTLs, only *OsLG1* was successfully cloned as the *SPR*3 locus [21]. *OsLG1* is a squamosa promoter binding protein (SBP) domain transcription factor that controls leaf tongue development in rice, had a significant influence on seed florescence and pollination (self-cross or outcross mode) [1, 24]. Further studies demonstrated that a single nucleotide polymorpho-6 (SNP6) in the 11 kb cis-regulatory region upstream of the transcriptional start site of *OsLG1* gene resulted in a compact panicle type in cultivated rice during rice domestication, but it did not change its expression in the leaf tongue, resulting in a compact panicle type and normal leaf tongue development in cultivated rice [1, 21].

Rice false smut (RFS) caused by *Ustilaginoidea virens* (*U. virens*), a unique flower-infecting fungal pathogen, has emerged as a serious grain disease in rice production worldwide. The disease not only causes a significant yield loss (up to 40%) but also produces various types of mycotoxins that contaminate rice grains and decrease the grain quality [28–30].

In recent years, although some studies have speculated that RFS is related to rice panicle traits, especially to the traits of large panicle, erect panicle and dense panicle [30, 31], but no specific related studies have been conducted to further prove this speculation. In this study, *SPR9*, a novel gene associated with spike development, was identified in the *spr9* mutant of R20-1 background. We analysed the genetic characteristics of the *spr9* mutant, mapped *spr9*, and found that the *spr9* gene contains a 1-bp deletion (T), leading to the loss of function of the *SPR9* gene. Interestingly, the *spr9* mutant altered the resistance to RFS. These results indicate that *spr9* has excellent potential application prospects in rice disease resistance breeding and spike-type improvement breeding.

## Methods

### Plant materials

*Indica* rice R20-1, which contains *Pigm-1* [32] and *japonica* Hui1586 [33], was cultivated by the Rice Research Institute, Fujian Academy of Agricultural Sciences, Fuzhou, China. In 2018, we planted approximately 600 plants of the M_1_ population treated by ethyl methanesulfonate (EMS) and approximately 10,000 plants of the M_2_ population, at the Sanya Experimental Station and Fuzhou Experimental Station, Fujian Academy of Agricultural Sciences, respectively. The M_2_ population was screened, and a spreading panicle mutant was obtained and named *spreading panicle 9* (*spr9*).

In the summer of 2020, the *spr9* mutant was crossed with rice varieties R20-1 and Hui1586 as pollen donors. F_1_ seeds were sown in the spring at the experimental station in Sanya, Hainan Province, and F_2_ seeds were harvested in 2021. The F_2_ seeds, the *spr9* mutant and R20-1 were planted at Fuzhou Experimental Station in Fujian Province in the summer of 2021. Hui1586 and the three knockout transgenic lines were planted at Fuzhou Experimental Station in Fujian Province in July 2022. The main agronomic traits, including plant height, panicle length, effective panicle number, spikelets per panicle, seed setting rate, 1000-grain weight, grain length and width, were investigated at the maturity stage. The segregation ratios of mutants versus the wild type were examined after maturity.

All plants were grown according to standard commercial procedures with spacing between rows of 13.3 and 26.4 cm, and field management was carried out according to normal field management practices.

### RFS inoculation and disease scoring

To evaluate the resistance to RFS disease between the *spr9* mutant and the wild type R20-1, inoculation methods were performed according to Song et al. with minor modifications [34]. Approximately 2 ml of the *U. virens* hyphae/conidia (10^6^ ml^− 1^) suspension in potato sucrose broth (PSB) was injected into a single rice panicle from the middle to the upper part at the late booting stage (3–5 days before heading) using an 18 gauge needle in a greenhouse. The liquid filled most of the panicle space. Three replications were carried out, three panicles of each individual plant were artificially inoculated. Inoculated plants were kept in a greenhouse at 27 °C with 90–100% RH for 7 days. Then, they were placed at 27 °C and 80% RH until RFS symptoms appeared.

According to the suggestion of a previous study [35], the incidence grade of RFS was divided into 6 grades, with no diseased grain per panicle as grade 0, 1 diseased grain per panicle as grade I, 2 diseased grains per panicle as grade II, 3–5 diseased grains per panicle as grade III, 6–9 diseased grains per panicle grain as grade IV, and > 10 diseased grains per panicle as grade V.

### Construction of the mapping population

The *spr9* mutant (*indica*) was crossed with Hui1586 (*japonica*) to obtain a mapping population. The F_2_ population was constructed by selfing the F_1_ population, and the mutant phenotypes of 1452 individual plants in the F_2_ population were selected for accurate mapping.

### Development of molecular markers

Insertion-deletion (InDel) markers were designed by manual comparison of genome sequences between *japonica* (cv. Nipponpare) and *indica* (cv. 93 − 11), and primers were designed to map the polymorphic regions of rice subspecies using Primer Premier 5.0.

### PCR amplification and molecular marker detection

Plant DNA and DNA amplification was performed by polymerase chain reaction (PCR) with minor modifications [36]. The PCR products were separated by 8% polyacrylamide denaturing gel electrophoresis, and the molecular markers were stained with silver [37].

### Bulked segregant analysis

Markers of target genes were identified by bulked segregant analysis. Leaf DNA of 20 mutant plants randomly selected from the F_2_ population were used for construction of a mutant DNA library. SSR markers distributed in the rice genome were used to amplify the *spr9* mutant DNA, and Hui1586 DNA was used as a control for linkage detection. The marker band of the mutant gene was the same as the marker band of the *spr9* mutant.

### Molecular mapping of the ***spr9*** gene

The band types of the mutant (*spr9 spr9*) and wild type (*SPR9 SPR9*) were denoted as 1 and 3, respectively; 2 was used to represent a heterozygote (*spr9 SPR9*). Linkage analysis was performed using MAPMAKER version 3.0 software [38], and the linkage map was basically the same as the linkage map reported previously [39].

First, 326 SSR markers were screened from the rice molecular map to study the polymorphisms of *spr9* and Hui1586. The results indicated that 253 pairs of primers showed polymorphism. Twenty plants with mutant phenotype and 20 plants like wild type selected from the F_2_ population, respectively, and linkage analysis of the *spr9* locus was performed using these 253 polymorphic markers. Second, to narrow the mapping region, we identified 1452 mutants from the F_2_ population of *spr9* × Hui1586. By comparing *Nipponbare* and the *indica* cultivar 93 − 11 (http://www.elabcaas.cn/rice/index.html), InDel markers in the open rice genome sequence were designed to predict the likelihood of polymorphism between *spr9* and Hui1586.

### Bioinformatics analysis

Candidate genes were predicted based on existing sequence annotation databases (http://rice.plantbiology.msu.edu/; http://www.tigr.org/). Clones were fixed on the target gene combination mark with the basic local alignment search tool (BLAST) in pairs (https://blast.ncbi.nlm.nih.gov/Blast.cgi?PROGRAM=blastn&PAGE_TYPE=BlastSearch&LINK_LOC=blasthome) for sequence alignment. The DNA sequences of *spr9* and *SPR9* were used for a complete alignment with Clustal X version 1.81.

### CRISPR mediated editing

The CRISPR-plant database and website were used to design gRNA spacer sequences with high specificity (Supplementary Table 1) [40]. A gRNA interval spanning the first exon of the gene was used to target the *SPR9* gene of Hui1586. The transformation and identification of the edited rice lines were performed by Wuhan Boyuan Technology Company. After obtaining the edited transgenic plants, PCR products of transgenic CRISPR-edited lines were sequenced to identify specific mutations [41]. Primers for the CRISPR/Cas9 study are listed in Supplementary Table 1.

### GFP fusion and subcellular localization

A *GFP-SPR9* fusion was constructed 35 S: SPR9-pSuper1300-GFP and transformed to *Agrobacterium tumefaciens* GV3101, which was used to infiltrate tobacco leaves, and the expression of GFP was observed by laser confocal microscopy (Zeiss 880) .

## Results

### Analysis of the main agronomic traits of ***spr9***

**Fig. 1:**
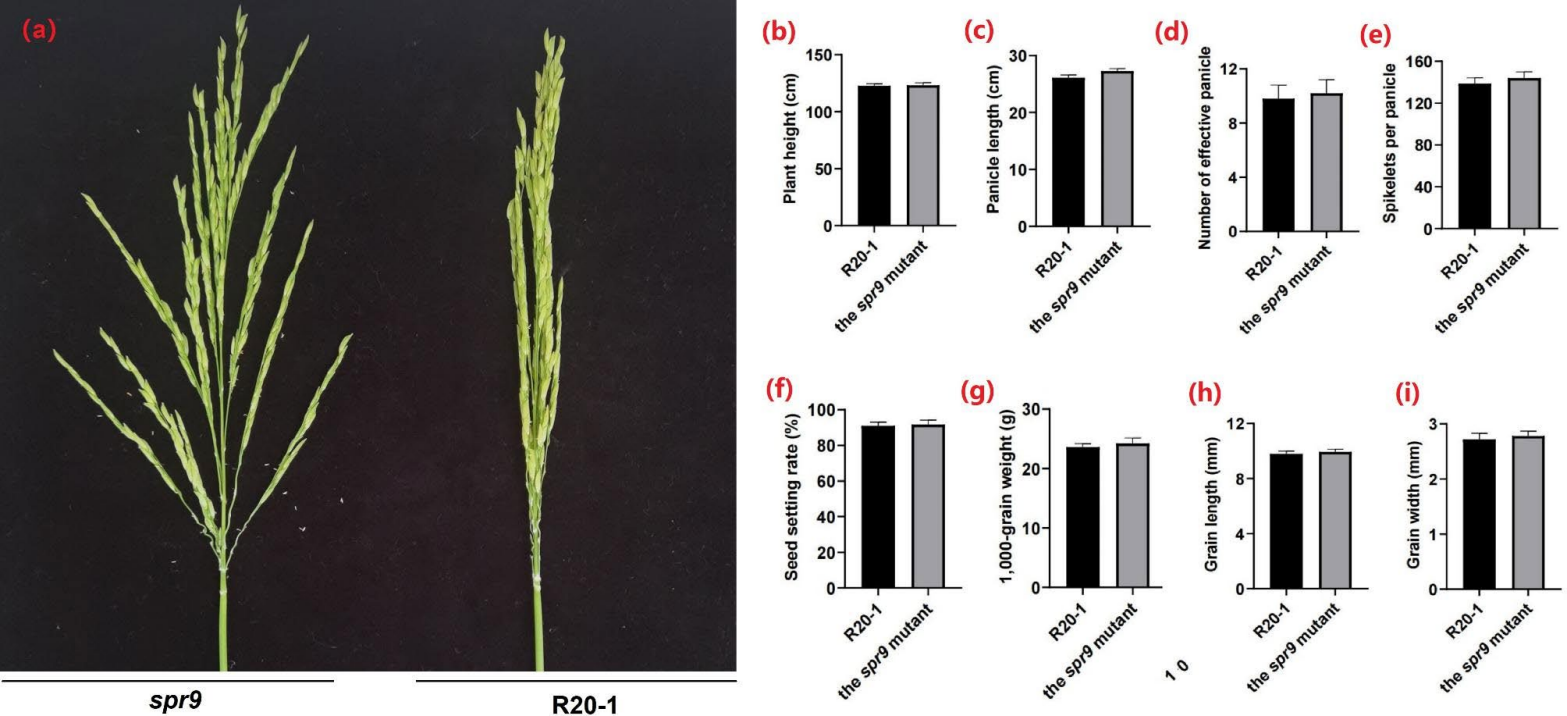
Phenotypic comparison of the *spr9* mutant and wild type R20-1. **a**: The *spr9* mutant showed the phenotype of spreading panicles compared with R20-1. **b**, **c**, **d**, **e**, **f**, **g**, **h**, and **i** indicate no differences in plant height, panicle length, number of effective panicles, spikelets per panicle, seed setting rate, 1,000-grain weight, grain length and grain width between R20-1 and the *spr9* mutant, respectively

To elucidate the regulatory genes involved in spike development in rice, we screened and obtained a *spr9* mutant with changes in spike traits in the R20-1 background, which displayed a spread panicle (Fig. 1a) and the corresponding wild type gene was named *Spreading Panicle 9* (*SPR9*). Phenotype comparisons between the *spr9* mutant and wild-type R20-1 are shown in Table 1. The results showed that there were no significant differences in plant height, panicle length, effective panicle number, number of grains per panicle, seed setting rate, 1000-grain weight, grain length or grain width between the *spr9* mutant and the wild type (Fig. 1b-I and Supplementary Table 2).

**Table 1:** Segregations of spreading panicle phenotype of the F_2_ populations produced from crossing of the *spr9* mutant and R20-1

Crosses	F_1_ phenotype	F_2_ population			*χ*^2^(3:1)	P
		Phenotype of R20-1	Phenotype of *spr9*	Total plants		
*spr9*/R20-1	Normal type	193	63	256	0.216*	> 0.9
R20-1/*spr9*	Normal type	228	77	305	0.124*	> 0.9

* Denotes the segregation ratio of normal plants to mutant plants complying with 3:1 at the 0.05 significance level

### Resistance analysis of RFS between the ***spr9*** mutant and R20-1

**Fig. 2:**
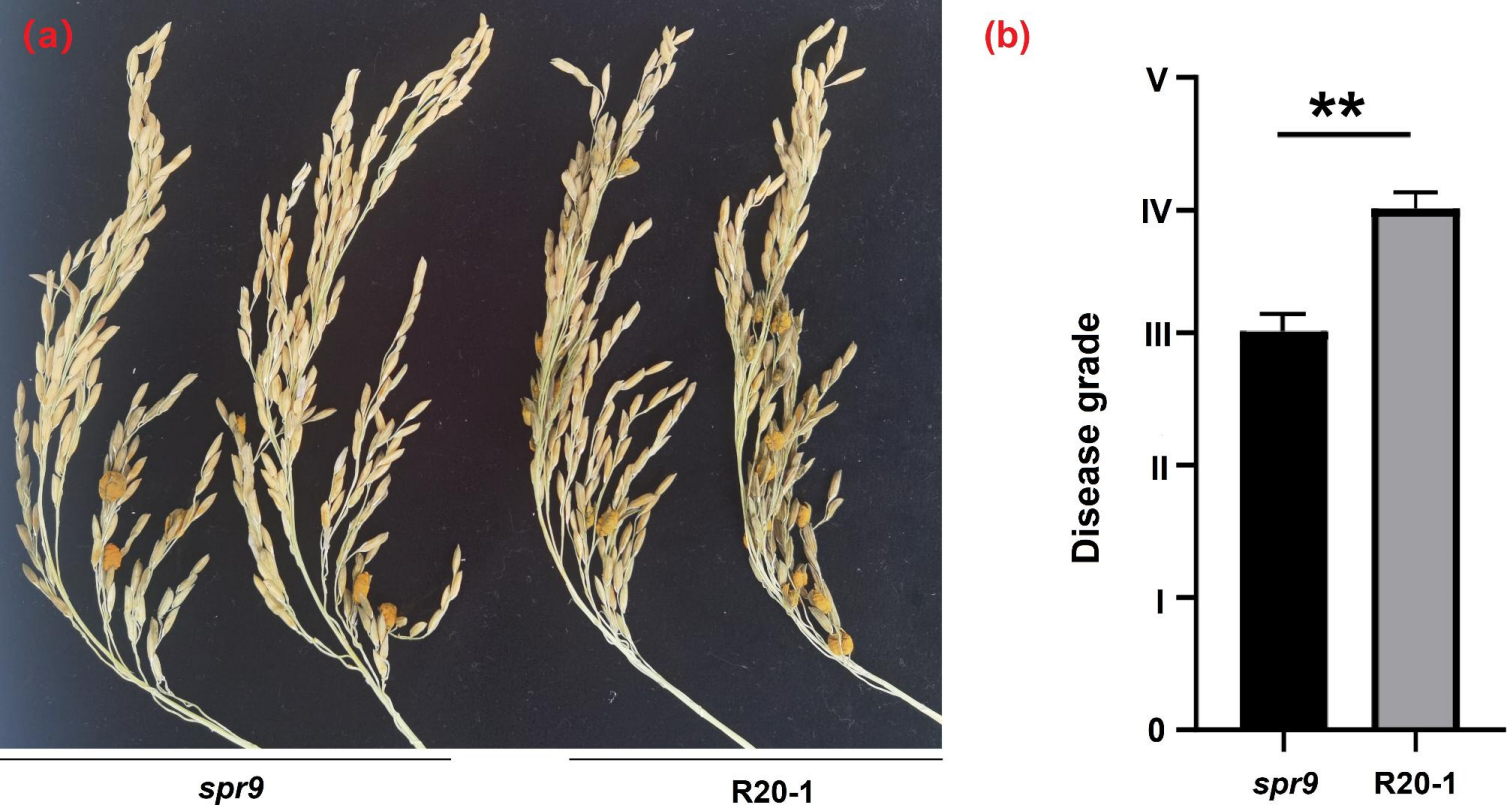
Disease symptoms of the *spr9* mutant and R20-1 infected by *U. virens*. **a**: Rice smut resistance of the *spr9* mutant and R20-1 plants using injected inoculation in a greenhouse. Three replications were carried out, each individual was artificially inoculated with *U. virens* in three panicles. Inoculated plants were kept in a greenhouse at 27 °C with 90–100% RH for 7 days. Then, they were placed at 27 °C and 80% RH until rice false smut symptoms appeared. **b**: The disease score of the *spr9* mutant is 3 and that of R20-1 is 4. The *spr9* mutant exhibited more resistance to rice smut than R20-1. Asterisks indicate statistical significance (*p <* 0.01) determined by Student’s *t-*test

A certain relationship was proposed between rice panicle type and the resistance level of RFS [30, 31]. To further compare whether there is a difference in resistance in RFS between the *spr9* mutant and R20-1, we inoculated the *spr9* mutant and R20-1 plants by manual injection, and each material was set up in triplicate. The experimental results showed that the disease score of the *spr9* mutant was 3 and that of R20-1 was 4, further suggesting that the *spr9* mutant exhibited more resistance to rice smut than the R20-1 control (Fig. 2a and b).

### Genetic analysis of the ***spr9*** mutant

To determine the genetic mode of *spr9* mutant, we generated two kinds of hybrid F_1_ plants by crossing *spr9* mutant with R20-1 and R20-1 with *spr9*, respectively. Both kinds of F_1_ plants showed same panicle phenotype as the wild type, indicating that the *spr9* was recessive heredity and independent of cytoplasm (Table 1). Then in the two F_2_ populations, we found that the panicle phenotype of plants as *spr9* mutant and R20-1 wild type fits a 1:3 segregation ratio (χ^2^ = 0.118～0.386, *P* > 0.5), implying a single gene controlled the *spr9* mutant phenotype. Together, these results indicate that the *spr9* mutant is genetically controlled by a single recessive gene.

### Preliminary molecular mapping of the ***spr9*** gene

**Fig. 3:**
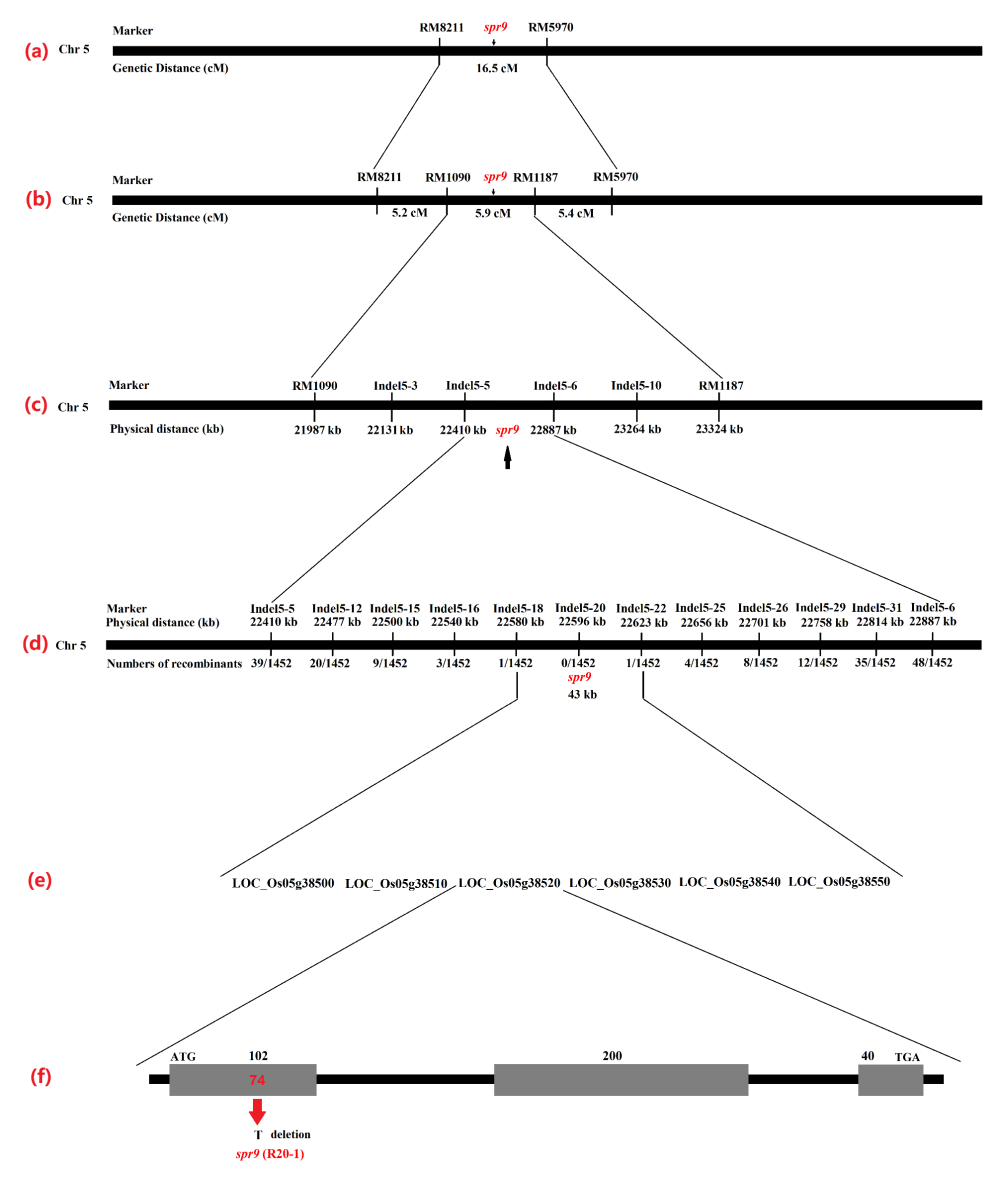
Physical maps and structural comparison of the *spr9* gene. **a**: Primary mapping of the *spr9* gene. The gene was mapped to the region between the markers RM8211 and RM5970. **b**: Further mapping of the *spr9* gene. The gene was mapped to the region between commonmarkers RM1090 and RM1187. **c**: Accurate mapping of the *spr9* gene. The *spr9* was mapped to the region between markers Indel15-5 and Indel15-6 selected from 10 newly developed InDel markers. **d**: Fine mapping of the *spr9* gene. The *spr9* was localized to a 43-kb region between the markers Indel15-18 and Indel15-22 selected from 21 newly developed InDel markers, and the recombinant number between the markers and target genes is indicated under the linkage map. **e**: Candidate genes in the 43-kb target region. f: *SPR9* has three exons, and *spr9* exhibits a 1 bp deletion in the first exon

To determine the gene underlying the *spr9* mutant, we further conducted a gene mapping population by crossing the *spr9* mutant with the japonica rice cultivar Hui1586. Total of 253 SSR markers showed polymorphism between *spr9* and Hui1586 were obtained. Among these polymorphic markers, RM8211, on chromosome 5, showed a complete co-segregation with mutant phenotype in the selected 20 F_2_ plants with mutant phenotype and 20 plants with wild type phenotype. Furthermore, 193 recessive plants from the F_2_ population were genotyped by RM8211 and RM5970, recombinant plants were found, verifying the linkage relationship between the marker and *spr9* mutant phenotype. Subsequently, we found one other polymorphic marker RM5970, located on chromosome 5, also showed linkage with *spr9* mutant phenotype. The recombinants identified from RM8211 all showed homozygous genotype as *spr9* mutant at the RM5970 locus, and the recombinants from RM5970 presented heterozygous or wild homozygous genotype at RM8211 locus, therefore, we preliminarily mapped the *spr9* gene into the region of RM8211 and RM5970. Thus, *spr9* was preliminarily located in a 16.5 cM region between SSR markers RM8211 and RM5970 on chromosome 5 (Fig. 3a).

### Fine mapping of the ***spr9*** gene

To localize the *spr9* gene in a smaller region, we constructed a genetic map between RM8211 and RM5970. Two pairs of polymorphic primers RM1090 and RM1187 were screened from the common primers between RM8211 and RM5970. Furthermore, 193 recessive plants from the F_2_ population were genotyped by RM1090 and RM1187, recombinant plants were found, verifying the linkage relationship between the marker and *spr9* mutant phenotype, *spr9* was located between the RM1090 and RM1187 molecular markers, and the distance between the two molecular markers was 5.9 cM (Fig. 3b). To further localize the *spr9* gene, 1452 recessive plants from the F_2_ population were genotyped by Indel5-3, Indel5-5, Indel5-6 and Indel5-10 (Table 2), recombinant plants were found, verifying the linkage relationship between the marker and *spr9* mutant phenotype. The localization results showed that the *spr9* gene was located between the molecular markers Indels 5–5 and 5–6, and the physical distance between the two markers was 477 kb (Fig. 3c; Table 2). To accurately locate the *spr9* gene, 1452 recessive plants from the F_2_ population were also genotyped by 10 polymorphic InDel markers Indel5-12, Indel5-15, Indel5-16, Indel5-18, Indel5-20, Indel5-22, Indel5-25, Indel5-26, Indel5-29 and Indel5-31 (Table 2), recombinant plants were found, verifying the linkage relationship between the marker and *spr9* mutant phenotype. The result showed 10 polymorphic InDel markers were detected 20, 9, 3, 1, 0, 1, 4, 8, 12 and 35 recombinant plants, respectively (Fig. 3d). Therefore, we accurately located the *spr9* gene between the Indel5-18 and Indel5-22 molecular markers, and the physical distance between them was 43-kb (Fig. 3d).

**Table 2:** InDel and SSR molecular markers used for fine mapping of the *spr9* gene

Marker	Sequence of forward primer	Sequence of reverse primer
RM8211	GTTTGGGAAGGAGGAATG	AAGTAGAAACGGCCAACAC
RM1090	GTTATAGCGCACCCTGGATG	GAACCGAAGGGACATGTGTG
Indel5-3	TGATTGATGTCTTCATCGTG	AACAAAAACCTCGATCTTGA
Indel5-5	GACATGACAAACGAAACACA	AGCAATCTCTAGGCAGTTGA
Indel5-12	AGCTCCTCTCCTCCTCCT	CAGGACCGGGAGTAAATTAT
Indel5-15	AAAGGACTGTTTCCCTGTTT	AAAGGGTGAAATCCTGATG
Indel5-16	TTTCCAAGTTCAAAATGCTT	TCAAAAAGGAAAAGATAAGG
Indel5-18	CACAACAACGAAAATACGAA	ACATGAACGAATGGTTGG
Indel5-20	ACCATTCTGATGTACGAAGC	AGCTTGCTGATCCAGTAGTC
Indel5-22	GGCCCCACTGATGTATTA	TTTGATATCATTCTTGTCCA
Indel5-25	GTACACGTGGCAGTCCAG	ACCTCTCGCCCTTGTGAT
Indel5-26	GTCGCAGAGGTACGTGAT	GACGTCGGGGGATACTTC
Indel5-29	CTTCGGCTTCATCTCCTC	CACATTTCTTCCTCCCCT
Indel5-31	GTCGACGTACATTCTCCATT	AGCCAGTAAAGCAAAGACTG
Indel5-6	CCCTCCGTACGGATACACAC	CTCTTCGGCTTCATCTCCTC
Indel5-10	TCGCATTGAGATTTGTGCAT	TCGTAACCACACTGCAACTG
RM1187	GTGGCTATGGCTACTGAGCC	CCGTTGTTGGTATCCAGGTC
RM5970	CCCATCTGGTTCACCTTCAC	AGGAGCAGCCTTTTGTCTTC

### Candidate gene analysis in the 43-kb region

The functions of six candidate genes were annotated (*LOC_Os05g38500*, *LOC_Os05g38510*, *LOC_Os05g38520*, *LOC_Os05g38530*, *LOC_Os05g38540* and *LOC_Os05g38550*) in this 43-kb region (Fig. 3e). According to the database annotation, the results showed that each of the six candidate genes had a corresponding full-length cDNA.

To find which gene is responsible for the mutant phenotype, we sequenced the above six genes in R20-1 and the *spr9* mutant, and the results showed that there was only a 1-bp deletion (T) (*LOC_Os05g38520*) between wild-type R20-1 and the *spr9* mutant (Fig. 3f). No difference were observed in the sequences of other five genes. Therefore, we hypothesized that *LOC_Os05g38520* corresponds to *SPR9*. Open reading fragment analysis showed that the *SPR9* gene (*LOC_Os05g38520*) had three exons and two introns (Fig. 3f).

### The ***spr9*** gene is responsible for the spread panicle phenotype

**Fig. 4:**
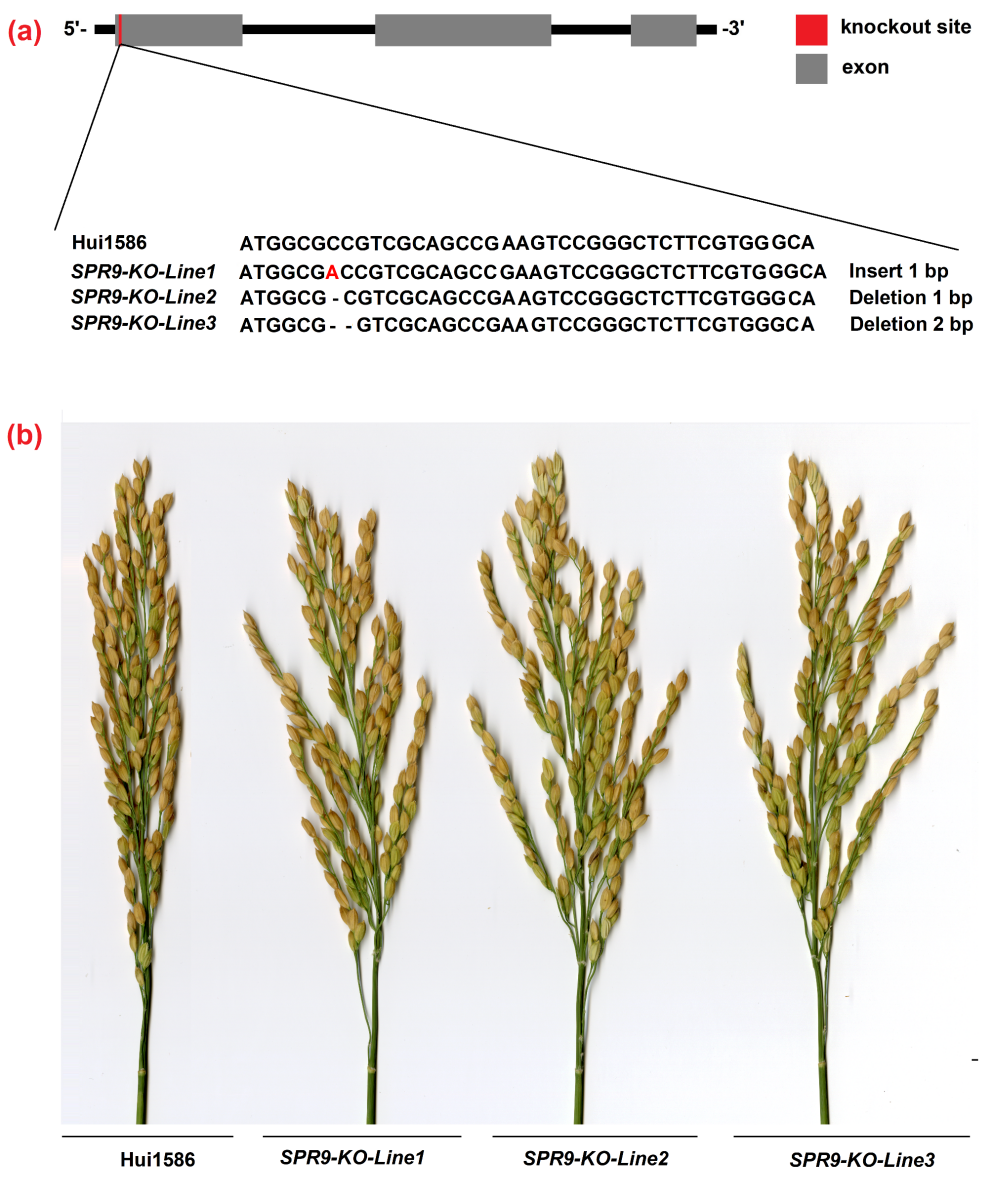
Knockout transgenic lines showed the phenotype of the *spr9* mutant. **a**: Three independent events (designated *SPR9-KO-Line1*, *SPR9-KO-Line2 and SPR9-KO-Line3*) were generated using the CRISPR/Cas9 system and verified by sequencing. **b**: Panicle differences between Hui1586 and three knockout lines. The three knockout lines generated by CRISPR/Cas9 all exhibit the phenotype of spreading panicles

To determine the phenotype of *spr9* in the *japonica* genetic background, we examined whether knockout of *SPR9* in the cultivar Hui1586 (*japonica*) would lead to the spread panicle phenotype. Using the CRISPR/Cas9 gene editing system, a sequence-specific guide RNA (sgRNA) was designed to knock out the *SPR9* gene. We obtained a total of three homozygotes from three independent knockout events and confirmed their presence of insertion and deletion mutations at the target sites by Sanger DNA sequencing (Fig. 4a). We then investigated and analysed the panicle characteristics of three homozygous lines after maturity and found that all three homozygous lines showed a panicle spread phenotype (Fig. 4b), which indicated that knockout of the *SPR9* gene in Hui1586 would lead to the spread panicle phenotype. In addition, analysis of other agronomic traits showed that there were no significant differences in plant height, panicle length, effective panicle number, number of grains per panicle, seed setting rate, 1000-grain weight, grain length or grain width between Hui1586 and three knockout transgenic lines (Supplementary Table 3). These results were consistent with the agronomic traits of the *spr9* mutant in the R20-1(wild-type). Taking together, we concluded that *spr9* gene was the causal gene for the spread panicle phenotype in the *spr9* mutant.

### Expression pattern and subcellular localizationof SPR9

**Fig. 5:**
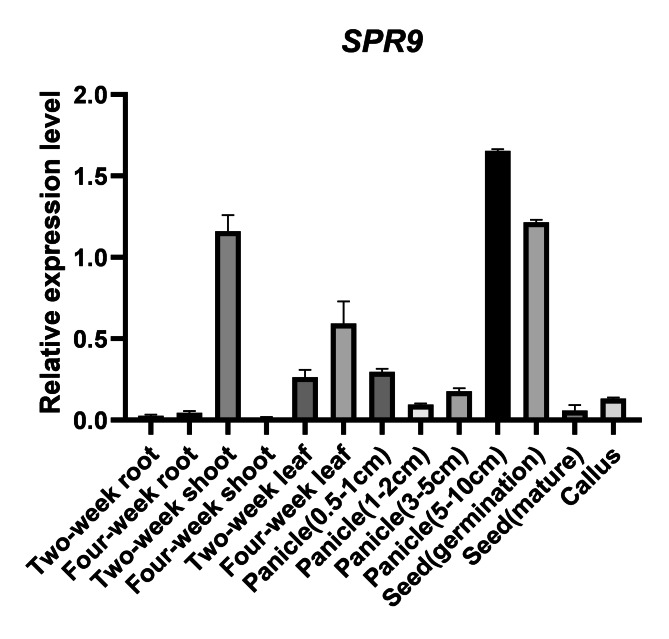
The expression patterns of *SPR9*. The expression patterns of *SPR9.* RNA samples were extracted from different tissues of Jiafuzhan, including roots, shoots, and leaves of two-, four- and six-week-old seedlings, spikelets of 0.5–1 cm, 1–3 cm, 3–5 cm, and 5–10 cm in length, germinating and mature seeds and callus. Data represent the mean and standard deviation of three biological replicates. Three technical replicates for each biological sample were used. The error bar represents the standard deviation (SD) of the value from three independent biological samples

To further understand the function of *SPR9*, reverse transcription- quantitative PCR (RT‒qPCR) was used to detect the expression patterns of *SPR9* at different developmental stages of rice (Primers are shown in Supplementary Table 1). The results showed that *SPR9* was expressed in all tissues tested here, including roots, shoots, and leaves of two- and four-week-old panicles of 0.5–1 cm, 1–3 cm, 3–5 cm, and 5–10 cm length, along with germinating and mature seeds and callus, but it was predominantly expressed in the seed (germination) and in panicles (5–10 cm length) (Fig. 5).

**Fig. 6:**
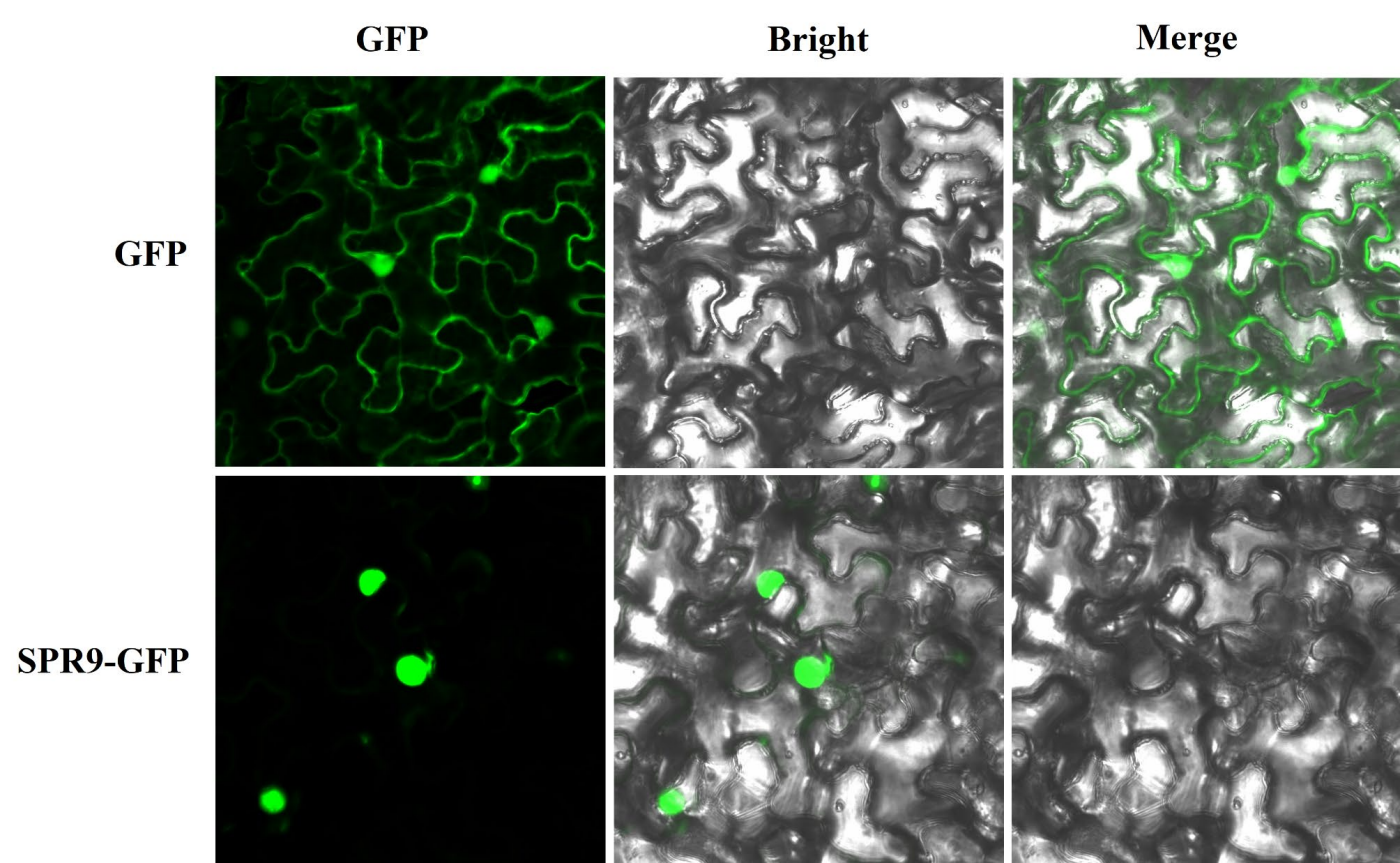
The subcellular localizationof SPR9. The localization of SPR9-pSuper1300-GFP in *Nicotiana tabacum* L. cells was observed by laser confocal microscopy. The results showed that SPR9-pSuper1300-GFP mainly expressed in the nucleus, Bar = 20 μm

In order to further analyze the localization of SPR9, we constructed 35 S: SPR9-pSuper1300-GFP vector (Primers are shown in Supplementary Table 1) and transformed to *Agrobacterium tumefaciens* GV3101. Three days after injecting tobacco leaves, the localization of SPR9 cells was observed by laser confocal microscopy (The Zeiss 880 confocal microscope). The results showed the localization of SPR9 in the nucleus (Fig. 6).

## Discussion

### ***SPR9 ***is a new spread panicle-related gene

The spread panicle trait is a typical mutant trait in rice, and there have been some reports on its gene mapping. To date, a total of 7 genes for spike traits have been reported, namely, *spr1* [22], *spr2* [23], *spr3*[24], *spr4* [25], *spr5*[26], *spr8* [27] and *OsLG1*[1]. Among these genes, *spr1* and *spr8* were controlled by a recessive nuclear gene, and other five sprouting trait genes were controlled by dominant genes. In this study, the *spr9* gene was identified as a recessive mutation. Through further comparison, no cloned spread panicle gene was found in this region, so we speculated that *spr9* was a new gene.

### Why does ***spr9*** show enhanced resistance to RFS?

*SPR9* encodes the 60 S ribosomal protein L36-2, which is a ribosome associated protein. However, why does *spr9* affect the disease resistance of rice? Studies have shown that posttranslational reprogramming is another fundamental regulatory pathway of plant immunity and specifically regulates translation in response to PAMP-triggered immunity induction [42]. Consistent with this result, previous transcriptomic studies of rice *Pi21-*silenced plants infected by *Magnaporthe oryzae* suggested that ribosomes were a third enrichment pathway compared to Nipponbare plants [43]. A recent study showed that translation was the most significantly enriched term in Gene Ontology (GO) analysis, while ribosomes were the most significantly enriched pathway in the Kyoto Encyclopaedia of Genes and Genomes (KEGG) analysis [33]. These results strongly indicated that the protein translation machinery was regulated during the response of rice to pathogens and is involved in rice immunity regulation. It was found that the ribosome protein gene *GhRPL18A-6* can regulate the expression of cell wall synthesis, lignin synthesis and other disease-resistant pathway genes in upland cotton, and improve the *Verticillium dahliae* resistance of transgenic cotton at various growth stages [44]. Based on the above relevant research results, we suspected that *SPR9* acts as a negative regulator of the immune response in rice. However, further biochemical experiments are needed to verify how *SPR9* is regulated by translation during immune activation in rice.

In recent years, studies on different crops have found that plant panicle architectures are closely related to plant disease resistance [45, 46]. In rice, it was found that the spike characters were related to the occurrence of RFS. The rice varieties with vertical close ear type are more susceptible to RFS, while the rice varieties with scattered ear type and long curved ear type are not [30, 31]. We speculated that the main reason for the relatively better resistance of spread or long-curved rice panicle types is that under the same environmental conditions, spread or long-curved rice panicle types have better permeability, short duration of high humidity conditions, and relatively low humidity. More importantly, studies have shown that the best condition for rice false smut is low temperature and high humidity [47, 48]. Therefore, this is consistent with the results of this study that the *spr9* mutants exhibit better disease resistance to RFS.

### The application prospect of the ***spr9*** gene in rice breeding

Many wild rice species have typical spread spikelets because the spread spikelets help them to improve the outcrossing seed setting rate and reproductive ability in the field to better adapt to the environment [49, 50]. At the same time, some studies have shown that the spread of panicle traits is often linked with some undesirable agronomic traits, such as shorter plant height, fewer tillers, fewer branches and stalks, lower yield, and stronger grain setting, but the grain quality is better[49, 50].

In this study, most importantly, our data indicated that the *spr9* mutant not only enhanced resistance to RFS but also did not affect the important agronomic traits of rice (Figs. 1 and 2 and Supplementary Table 2). Together, the results indicate that *SPR9* has good application prospects in future rice disease resistance and improved panicle breeding. For example, to improve the outcrossing rate of male sterile lines, we can transfer *spr9* into sterile lines. At the same time, to improve rice smut resistance to a certain extent, we transferred *spr9* into restorer lines.

## Conclusion

In this study, a novel *SPR9* gene was mapped and identified as a ribosomal protein coding gene. Then, CRISPR/Cas9 knockout experiments confirmed that the *SPR9* gene is responsible for the spreading panicle phenotype of the *spr9* mutant. Importantly, the *spr9* mutant was found to improve resistance to RFS without affecting major agronomic traits, indicative of potential applications of *spr9* in broader breeding programs. Taken together, our results revealed that the *spr9* gene has good application prospects in future rice disease resistance and improved panicle breeding.

## Electronic supplementary material

Below is the link to the electronic supplementary material.

Supplementary Material 1

Supplementary Material 2

Supplementary Material 3

## Data Availability

The datasets used and/or analysed during the current study are available from the corresponding author on reasonable request. The genome sequence of *LOC_Os05g38520* (*SPR9*) can be found in the NCBI database (http://www.ncbi.nlm.nih.gov/), and the number of GenBank is AK058918.
