# Supplementary material for: SPR9 encodes a 60 S ribosomal protein that modulates panicle spreading and affects resistance to false smut in rice (Oryza sativa. L)
Source: BMC Plant Biol. 2023 Apr 20;23:205. doi: 10.1186/s12870-023-04172-4 (PMC10116690; doi:10.1186/s12870-023-04172-4)
Supplement: Supplementary file 1 — Supplementary Material 1 [file 12870_2023_4172_MOESM1_ESM.docx]

**Supplementary Table 2.** Comparison of the main agronomical traits between the *spr9* mutant and R20-1

| Traits | R20-1 | *spr9* mutant |
| --- | --- | --- |
| Plant height (cm) | 122.6 ± 2.01 | 123.2 ± 2.12 |
| Panicle length (cm) | 26.1 ± 0.42 | 27.3 ± 0.44 |
| Number of effective panicles | 9.8 ± 1.08 | 10.2 ± 1.12 |
| Spikelets per panicle | 139.1 ± 5.21 | 143.8 ± 5.98 |
| Seed setting rate (%) | 90.94 ± 2.12 | 91.74 ± 2.65 |
| 1,000-grain weight (g) | 23.63 ± 0.63 | 24.31 ± 0.82 |
| Grain length (mm) | 9.81 ± 0.21 | 9.94 ± 0.20 |
| Grain width (mm) | 2.72 ± 0.11 | 2.78 ± 0.09 |

Note: The data was derived from the trial that was performed at the Fuzhou experimental station in April 2021. **P<0.05* and ***P<0.01* for the differences between R20-1 and *spr9* mutant.
