# Supplementary material for: SPR9 encodes a 60 S ribosomal protein that modulates panicle spreading and affects resistance to false smut in rice (Oryza sativa. L)
Source: BMC Plant Biol. 2023 Apr 20;23:205. doi: 10.1186/s12870-023-04172-4 (PMC10116690; doi:10.1186/s12870-023-04172-4)
Supplement: Supplementary file 2 — Supplementary Material 2 [file 12870_2023_4172_MOESM2_ESM.docx]

**Supplementary Table 3.** Comparison of the main agronomical traits between Hui1586 and three knockout transgenic lines

| Traits | Hui1586 | *SPR9-KO-Line1* | *SPR9-KO-Line2* | *SPR9-KO-Line3* |
| --- | --- | --- | --- | --- |
| Plant height (cm) | 86.22 ± 1.82 | 87.62 ± 1.89 | 86.62 ± 1.76 | 87.22 ± 1.88 |
| Panicle length (cm) | 19.84 ± 1.21 | 19.88 ± 1.17 | 20.10 ± 1.28 | 19.95 ± 1.23 |
| Number of effective panicles | 10.32 ± 1.26 | 10.45 ± 1.34 | 10.16 ± 1.18 | 10.26 ± 1.09 |
| Spikelets per panicle | 150.24 ±3.88 | 151.76 ±4.01 | 149.76 ±3.98 | 151.16 ±4.09 |
| Seed setting rate (%) | 90.74 ± 1.96 | 88.22 ± 1.66 | 89.14 ± 186 | 88.01 ± 2.01 |
| 1,000-grain weight (g) | 25.32± 0.58 | 26.01± 0.71 | 25.82± 0.63 | 26.06± 0.72 |
| Grain length (mm) | 8.31 ± 0.24 | 8.24 ± 0.26 | 8.41 ± 0.22 | 8.32 ± 0.16 |
| Grain width (mm) | 3.69 ± 0.07 | 3.70 ± 0.08 | 3.71 ± 0.09 | 3.70 ± 0.08 |

Note: The data was derived from the trial that was performed at the Fuzhou experimental station in October 2022. **P<0.05* and ***P<0.01* for the differences between Hui1586 and three knockout transgenic lines.
