# Supplementary material for: SPR9 encodes a 60 S ribosomal protein that modulates panicle spreading and affects resistance to false smut in rice (Oryza sativa. L)
Source: BMC Plant Biol. 2023 Apr 20;23:205. doi: 10.1186/s12870-023-04172-4 (PMC10116690; doi:10.1186/s12870-023-04172-4)
Supplement: Supplementary file 3 — Supplementary Material 3 [file 12870_2023_4172_MOESM3_ESM.doc]

**Supplementary Table 1. Primer sequences used for synthesizing gRNA spacers and genotyping CRISPR-edited mutants**

| Name | sequence 5’→3’ | Purpose |
| --- | --- | --- |
| gRNAs-*SPR9* | GGCCGTCGCAGCCGAAGTCC | CRISPR/CAS9 |
| *SPR9-*F1 | CCACAAGCCCACAACAAAGG | Screening of lines |
| *SPR9-*R1 | AAACACGGACAAGCATCACC | Screening of lines |
| *SPR9-*qRT-PCR-F | CTCTTCGTGGGCATCAACA | RT-qPCR |
| *SPR9-*qRT-PCR-R | GGAGCAAATCCAGCAACCT | RT-qPCR |
| *UBIQUITIN*-F | AACCAGCTGAGGCCCAAGA | Reference gene |
| *UBIQUITIN*-R | ACGATTGATTTAACCAGTCCATGA | Reference gene |
| SPR9-pSuper1300-GFP-F | CTGCAGGGGCCCGGGGTCGAC ATGGCGCCGTCGCAGCCGAAGT | Subcellular localization |
| SPR9-pSuper1300-GFP-R | CCCTTGCTCACCATGGTACC TTTCTTCTTCTCGGTGTGAGC | Subcellular localization |
